# Revisit Population-based and Family-based Genotype Imputation

**DOI:** 10.1038/s41598-018-38469-4

**Published:** 2019-02-12

**Authors:** Ching-Ti Liu, Xuan Deng, Virginia Fisher, Nancy Heard-Costa, Hanfei Xu, Yanhua Zhou, Ramachandran S. Vasan, L. Adrienne Cupples

**Affiliations:** 1 Department of Biostatistics, Boston University School of Public Health, Boston, MA 02118 USA; 2 Department of Medicine, Boston University, Boston, MA 02118 USA; 3 Framingham Heart Study, Framingham, MA 01702 USA

## Abstract

Genome-Wide Association (GWA) with population-based imputation (PBI) has been successful in identifying common variants associated with complex diseases; however, much heritability remains to be explained and low frequency variants (LFV) may contribute. To identify LFV, a study of unrelated individuals may no longer be as efficient as a family study, where rare population variants can be frequent in families. Family-based imputation (FBI) provides an opportunity to evaluate LFV. To compare the performance of PBI and FBI, we conducted extensive simulations, generating genotypes using SeqSIMLA from various reference panels for families. We masked genotype information for variants unavailable in Framingham 550 K GWA genotype data in less informative subjects selected by GIGI-Pick. We implemented IMPUTE2 with duoHMM in SHAPEIT (Impute2_duoHMM) for PBI, MERLIN and GIGI for FBI and PedBLIMP for a hybrid approach. In general, FBI in both MERLIN and GIGI outperformed other approaches with imputation accuracy greater than 0.99 for the squared correlation and imputation quality scores (IQS) especially for LFV, although imputation accuracy from MERLIN depends on pedigree splitting for larger families. PBI performed worst with the exception of good imputation accuracy for common variants when a closely ancestry matched reference is used. In summary, linkage disequilibrium (LD) information from large available genotype resources provides good imputation for common variants with well-selected reference panels without requiring densely sequenced data in family members, while imputation of LFV with FBI benefits more from information on inheritance patterns within families yielding better imputation.

## Introduction

Genotype imputation derives from statistical inference of genotypes that are not directly assayed. In recent decades, genotype imputation has been commonly used in genome-wide association studies (GWAS) since it provides the same set of single nucleotide polymorphisms (SNPs) to collaborating studies, thereby enabling researchers to combine genotype-phenotype association results from participating cohorts using different genotyping platforms. Thus, genotype imputation efficiently increases the effective sample size and improves statistical power to detect disease variants with moderate effects. Genotype imputation strategies can be broadly classified into population-based methods, which use population linkage disequilibrium (LD) information, and family-based methods, which use inheritance information within pedigrees.

Population-based methods impute unobserved SNPs using a reference panel of subjects with complete observations on a more comprehensive set of SNPs. These reference panels, such as HapMap and 1000 Genomes^1,2^, represent most common genetic variation in human populations. LD or correlation between nearby SNPs is used to predict unobserved genotypes in a study sample with a sparser set of variants. For example, IMPUTE2^3^ was developed as a population-based imputation method that improves upon previous approaches by using both the reference and study samples to inform haplotype phasing at observed markers. SHAPEIT2 duoHMM^4^ further improves upon phasing in family-based studies with a hidden Markov model for identity by descent (IBD), with results incorporated into an IMPUTE2 analysis.

The era of GWA with population-based imputation has been highly successful in identifying common variants associated with many diseases and their risk factors. Nevertheless, GWAS have not explained all genetic variation in association with the diseases and risk factors that affect large numbers of U.S. individuals. Much genetic variation remains to be explained^5^ and larger samples are needed to identify variants with weaker effects and/or lower frequency. Whereas we once thought that only a few genes/variants might be associated with disease, we have now reconfirmed the paradigm that the genetic determinants of many complex diseases derive from a polygenic model comprising many genetic variants with small effects^6–9^.

To identify low frequency variants (LFV), a population study of unrelated individuals is no longer an efficient study design. Family studies offer an effective, and less expensive, option to pursue such variation with adequate power, as rare population variants will be more frequent in families where a founder has the variant. Further, multiple rare variants in a gene in several families may act in a similar fashion on traits. Hence, linkage studies or collapsed rare variant analyses in such families may have improved power compared to studies of unrelated individuals and also provide identification of those variants that are segregating through the family with the trait^10^.

Thus, imputation in family studies provides an opportunity to evaluate rarer variants and their effects on disease and associated risk factors. Several methods have been proposed for situations where some family members have been genotyped on a dense set of markers, and other members have only sparse marker genotypes measured. For example, Merlin^11,12^ uses pedigree structure to identify inheritance vectors within a family, then propagates genotypes at high-density markers observed in a subset of individuals to others in the pedigree. GIGI (Genotype Imputation Given Inheritance)^13^ uses a two-stage procedure to infer inheritance vectors at sparse markers, then uses MCMC sampling to estimate genotypes of a dense marker set. With the growing availability of whole genome sequences, family-based imputation may provide improved strategies for imputing rare variants. In this paper, we compare two family-based imputation (FBI) methods to population-based imputation (PBI) as well as a hybrid version that combines FBI and PBI.

## Materials and Methods

**Figure 1:**
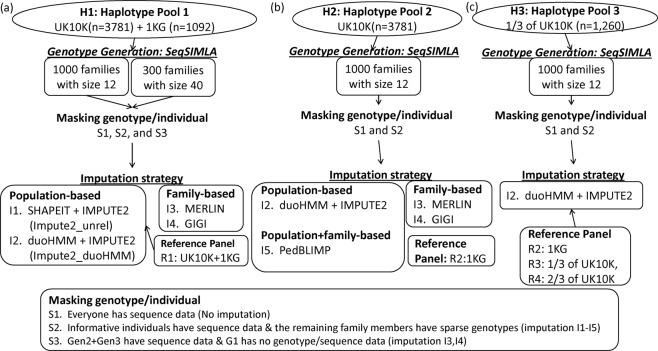
Flow chart of the study design.

We carried out a series of simulations to evaluate the performance of PBI and FBI strategies in several scenarios. Figure 1 summarizes our study design and simulation procedures. We simulated sequence data and masked some genotypes to evaluate the imputation approaches. For PBI, we considered several scenarios using dense reference panels from UK10K and 1000 G. The identification of genetic variants for the sparse backbone panel that would be used for imputation was based on the array variants from the 250 K Nsp, 250 K Sty Affymetrix and the 50 K gene centric Affymetrix arrays that were observed in the Framingham Heart Study. All imputation methods were restricted to diallelic single nucleotide variants (SNVs). FBI used informative individuals in families with sequence data to impute variants missing in other members within the same family. Family members with missing variants have backbone variants or no genetic data. We focused on 5 imputation strategies: I1) SHAPEIT without pedigree information for phasing followed by IMPUTE2; I2) SHAPEIT with duoHMM for phasing that takes into account pedigree information followed by IMPUTE2; I3) MERLIN for estimating IBD followed with genotype imputation; I4) GIGI with pre-calculation of inheritance vectors in MORGAN; and I5) PedBLIMP^14^ using LD information from external panel data and IBD information from internal family data.

### Phasing and Imputation Strategies

We estimated unknown haplotypes from typed genotypes (e.g. GWA array variants), known as phasing, for each subject with SHAPEIT (both with and without using the duoHMM feature) and followed by genotype imputation for PBI implemented via IMPUTE2^4,15,16^. For FBI, we implemented two approaches for imputation. One is GIGI, where imputation for each member of a family follows estimation of the pattern of genetic transmission within the family based on the observed genotype data^17^. We used MORGAN for this estimation of inheritance vectors^18^. The second FBI approach was implemented in MERLIN, which performs estimation of inheritance vectors for families and does not require an additional step for haplotype estimation^12^. Additionally, we considered a hybrid approach, PedBLIMP, which calculates the covariance matrix for LD information from an external panel and then estimates IBD for each locus to obtain the locus specific relatedness matrix^14^. We briefly describe each approach.

### Haplotype estimation and Inheritance vectors estimation

Haplotype phasing and inheritance vector estimation are essential steps in PBI and FBI. We used SHAPEIT version v2-790 to pre-phase prior to PBI. SHAPEIT estimates haplotypes from directly measured genetic variants (e.g. array-based genotypes) before imputing genotypes for those un-typed variants from a denser reference haplotype panel. This pre-phasing step improves the imputation speed since the haplotype estimation needs to be done only once. We considered two scenarios in haplotype phasing. One ignored relatedness information within families and another improved phasing by incorporating family information through a hidden Markov model (duoHMM)^4^.

For FBI, we used PLINK 1.9 to perform LD-based SNP pruning, with window size of 50 SNPs and shifted by 5 SNPs at each step, to construct a list of SNPs in approximate linkage equilibrium with pairwise LD r2 < 0.2 from available directly genotyped data. The SNPs in linkage equilibrium identified by this analysis were selected from simulated data and used to estimate the number of alleles shared IBD among relatives in a pedigree in MERLIN. We used the same SNPs to infer inheritance vectors summarizing the pattern of genetic transmission through pedigrees, based on estimated genetic locations of meiotic events with the gl_auto routine in MORGAN 3.1. The same process was used for PedBLIMP.

### Large Pedigree Splitting

An important property of MERLIN is that it can only evaluate smaller pedigrees. For the scenario where pedigrees exceed the computational limit for imputation in MERLIN, we used a pedigree splitting algorithm previously described^19^. Briefly, the algorithm used kinship coefficients for splitting and trimming each large pedigree into multiple smaller sub-pedigrees of a user pre-specified size (in our case, maxbits = 18 or 20 and bit size = 2 * # non-founders – # founders – # un-genotyped founder couples). The basic steps started with clustering un-genotyped individuals with their un-genotyped first-degree relatives and then constructing sub-pedigrees based on these clusters and their genotyped relatives. The same procedure applied to the sub-pedigrees until their bit sizes meet the pre-selected size. Essentially, each sub-pedigree produced was centered on one or more un-genotyped individuals with as many closely related genotyped relatives as possible. In the case where an individual was included in multiple sub-pedigrees, imputation was performed using one sub-pedigree at a time and the imputations with the best quality for an individual were used for further analysis.

### Genotype Imputation

We used IMPUTE2 v2.3.0 to impute genotypes for untyped (masked) variants using several reference panels^3^. IMPUTE2 can use either unphased genotype data or phased haplotype data to estimate unobserved genotypes. However, pre-phasing lowers the computational time significantly; hence we phased our data first with SHAPEIT before conducting imputation with IMPUTE2.

For FBI, we consider three implementations: MERLIN v1.1.2, GIGI v1.04, and PedBLIMP v0.2.0. MERLIN uses dense genotype data from a subset of individuals in a pedigree to infer missing genotypes in remaining relatives, who may or may not have sparse genotype data. It first uses low-density genotypes in all family members to estimate the number of alleles shared IBD in a pedigree. Then, with IBD and high-density genotype data in a subset of family members, unobserved high-density genotypes for remaining members can be imputed. The imputation implemented in MERLIN uses the Lander-Green algorithm^20^, in which posterior probabilities of the three possible genotypes at each SNV are calculated. MERLIN imputation can be very rapid and efficient in small pedigrees; however, the complexity of Lander-Green algorithm increases exponentially with pedigree size. Pedigree size 12 used in our study is sufficiently small for this algorithm^21^. For pedigree size 40, sub-pedigrees were constructed as described above.

GIGI uses a two-step procedure to impute dense genotypes in pedigrees where only a few members have high-density genotype or sequence data available, and most members have been genotyped on a sparse set of “framework” markers in linkage equilibrium. After summarizing the pattern of genetic transmission through the pedigree as inheritance vectors, using MORGAN, the second step uses the inferred inheritance vectors and all available dense marker sequence data to impute unobserved genotypes by estimating locations of meioses and filling in variants between framework markers.

PedBLIMP^14^ extends the population-based method BLIMP^22^, which uses conditional multivariate normal moments to impute genotypes with LD information from an external panel, by additionally taking the pedigree structure or IBD information into consideration. PedBLIMP first builds a precomputed shrinkage covariance and mean of genotypes from the reference panel data, and then imputes SNP data using the expected relatedness matrix calculated from the pedigree structure.

### Study Design and Genotypic Data Simulation

Figure 1 displays our study design including genotype data simulation, genotype masking scenarios and imputation strategies.

### Sequence Simulation

To compare imputation strategies, we simulated dense DNA sequence data on a selected region on chromosome 20 using SeqSIMLA^23^ to generate genotype data for family members (Supplemental Fig. 1), based on the available haplotype pool. Specifically, we considered the following haplotype pools: H1: The combined haplotype information from the UK10K (http://www.uk10k.org/, n = 3,781) and the 1000 Human Genome Metropolitan Panel (1000 G, n = 1,092); H2: Haplotype information from UK10K only; H3: one-third of haplotype information randomly selected from UK10K (n = 1,260) (Fig. 1). We first simulated 1000 families of size 12 (total 12 K subjects) with identical pedigree structure. We also simulated 300 families of size 40 (total 12 K subjects). Our results primarily focused on the scenario with family size 12.

The region from 1965933 to 4025750 (hg19) on chromosome 20 was selected as it contains variants with a full range of minor allele frequency (MAF) from 0.028% to 49.98%. The majority of variants were less frequent: the proportions of variants with MAF >5%, 1–5% and <1% were 19.3%, 9.2% and 71.4% in the H1 pool, respectively. In addition, we selected this region to be large enough to contain various LD structures, a strong LD block and a weak/mild LD block, and small enough to handle the multiple simulations in the current study. There are 26,308 variants in total within this region. To mimic reality, we assumed that dense genotypes were observed only for ‘informative’ family members. Informative family members were identified with GIGI-Pick^13^, providing a guideline to sequence a minimal number of individuals to deduce other family members’ sequence through imputation. For the remaining family members, we masked the simulated genotype information except for a set of sparse variants available in commonly used genotype array platforms. Supplemental Fig. 1 identifies the ‘informative’ subjects used in our simulation setting.

### Masking genotypic information: Imputation Scenarios

We considered three scenarios for selecting which members of the pedigree have dense genotype information from the sequence data (Fig. 1). The first scenario (S1) assumed that everyone has sequence data, an unlikely circumstance. There are 26,308 variants in total within the pre-selected region from our simulation. This scenario served as a benchmark to compare with others. The second scenario (S2) assumed that only informative individuals have dense sequence data and the remaining family members have sparse genotype (or backbone) data. The identification of the sparse genotypes was based on the genotype information in the Framingham Heart Study (FHS) SHARe project, for which genotyping was conducted using approximately 550,000 SNPs (Affymetrix 250 K Nsp and 250 K Sty mapping arrays plus Affymetrix 50 K gene-centric supplemental array). Specifically, there were 447 variants within the region of interest, 136 of which remained after LD pruning. The third scenario (S3) was designed to evaluate the performance of imputing the ‘top’ generation, specifically the first generation here, assuming that sequence data was available for all their descendants and relatives (the second generation and their spouses and the third generation). Such a circumstance occurs when the members at the top of a pedigree in a sample of families are dead and have no genotype data.

For reference panels for IMPUTE2, we considered: R1: combined haplotype information from 1000 G and UK10K; R2: 1000 G only; R3: one-third of UK10K without overlapping with H3 haplotype pool; R4: two-thirds of UK10K from the complementary set of the H3 haplotype pool. For PedBLIMP, we used R2:1000 G as the reference panel.

### Evaluation of Imputation Performance

Several measures of imputation performance are available to evaluate imputation quality, for example, concordance, squared correlation and imputation quality score (IQS) with the true sequence genotypes. Concordance between imputed and true simulated genotypes uses the best guess genotype and thus, depends on the threshold of the posterior probability for the best guess. As a result, it overestimates the accuracy for rare or less common variants due to random chance of concordance. In addition, by using the best guess and ignoring the posterior probability for the best guess, it ignores the uncertainty of imputed genotypes that is reflected in the dosage. Prior literature has shown lower power in association analyses that use the best guess compared to those methods that use genotype dosage or take uncertainty of imputed genotypes into consideration^24^. Therefore, we used the squared correlation and IQS as measures of imputation quality for comparisons among different imputation methods.

### Squared Correlation (r^2^)

The squared correlation estimate for each SNP is the squared Pearson correlation between the masked true genotypes, which take values of 0, 1, 2, and the imputed dosages, which take values in the range [0, 2]. Squared correlation estimates are not very reliable measurements of the accuracy for rare variants. For example, the squared correlation of a SNP with low minor allele frequency is unmeasurable because either the true genotypes or the imputation dosages might be identical for all individuals. We used the squared correlation here to compare the different imputation approaches and to contrast with the IQS.

### Imputation quality score (IQS)

We also consider the imputation quality score (IQS), originally inspired by Cohen’s Kappa statistic and developed by Lin *et al*.^25,26^. IQS measures the agreement between two classifications, adjusting for chance agreement. The calculation of IQS requires the posterior probabilities of imputed genotypes. Denote *n*_*ij*_ as the number of individuals with true genotype *i* and imputed additive genotype *j*, *n*_*.j*_ as the total number of individuals with imputed genotype *j*, *n*_*i*_ as the total number of individuals with true genotype *i* and *n*_.._ is the total sample size. IQS is calculated by subtracting the chance agreement *P*_*c*_ from the observed agreement *P*_*o*_ and dividing by the maximum possible value of the numerator: $IQS=\frac{{P}_{o}-{P}_{c}}{1-{P}_{c}}\,$ where ${P}_{O}=\frac{{\sum }_{i}{n}_{ii}}{{n}_{\mathrm{..}}}$ and ${P}_{c}=\frac{{\sum }_{i}{n}_{i.}{n}_{.i}}{{n}_{\mathrm{..}}^{2}}$.

IQS incorporates the uncertainty of imputation by using posterior probabilities for genotypes instead of the best-guess genotype, thus avoiding potential bias caused by the selection of a threshold for posterior probability. IQS is useful for rare variants because it accounts for allele frequency and adjusts for chance agreement. IQS can be computed using dosages, yielding more information on imputation quality. Details of IQS can be found in Lin *et al*.^26^.

## Results

### Imputation Accuracy

**Figure 2:**
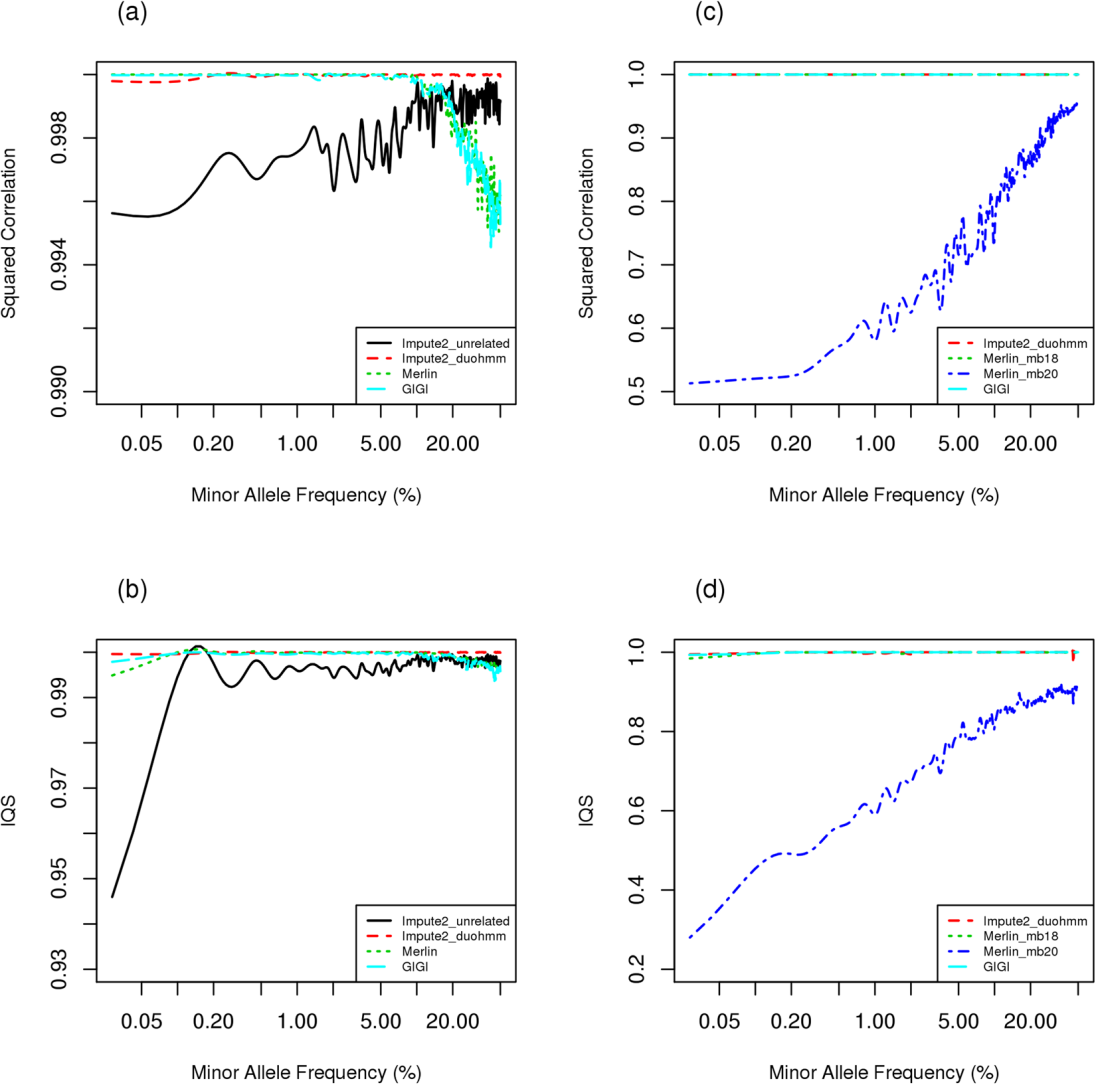
Comparison of imputation accuracy with squared correlation and imputation quality score among various imputation strategies under the scenario that all informative people have dense genotypes for family-based imputation and all individuals have sparse genotype as backbone for population-based imputation, using genotypes simulated from haplotype pool 1 (UK10K + 1000 G sample). The left panel, (**a,b**), of the figure shows the imputation accuracy for the scenario with 1000 families of size 12; the right panel, (**c,d**), of the figure shows the imputation accuracy for the scenario with 300 families of size 40. For family size of 40, two different pedigree-splitting methods (Merlin_mb18 and Merlin_mb20) are considered based on maxbits of 18 or 20 for the imputation using Merlin. The x-axis in the figures is on a logarithmic scale.

**Figure 3:**
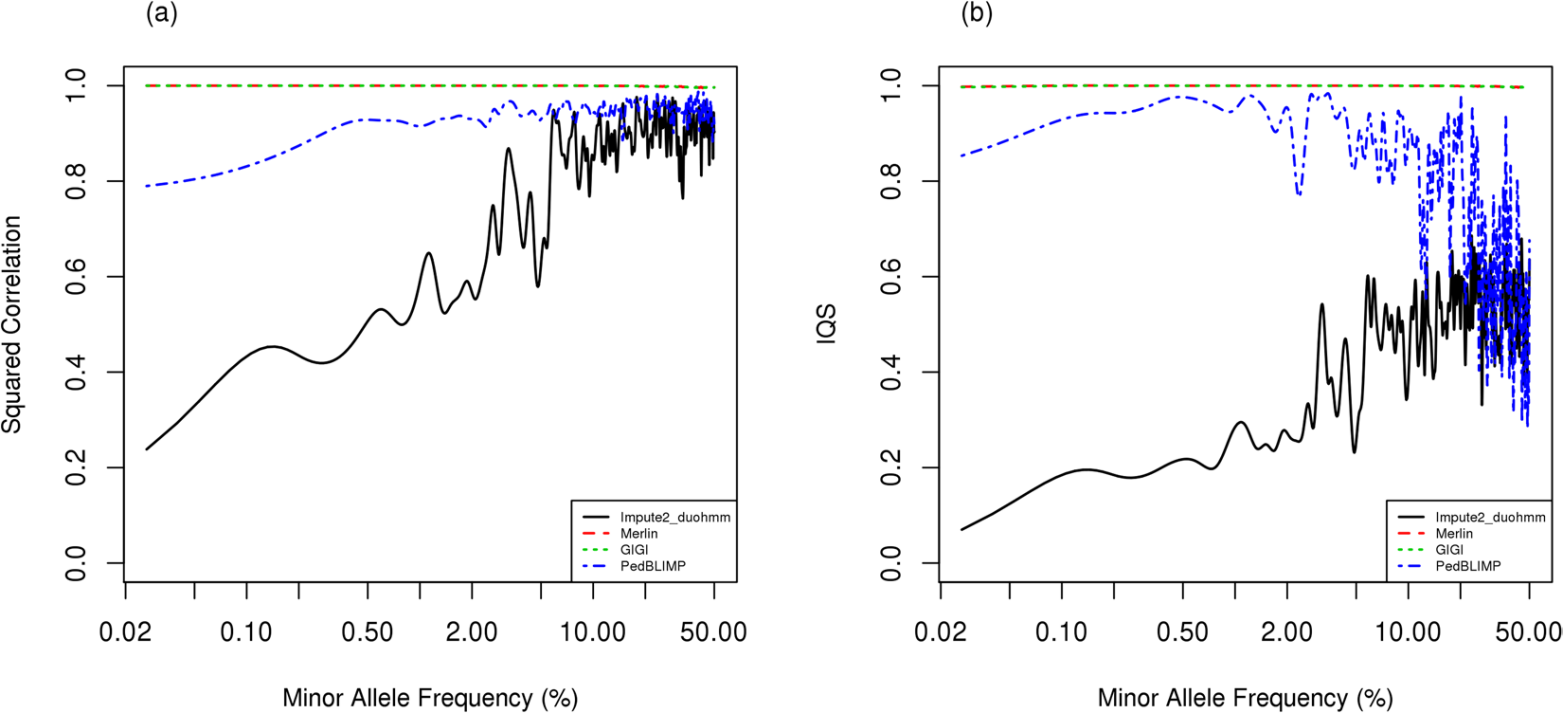
Comparison of imputation accuracy with squared correlation, (**a**) and imputation quality score, (**b**) among various imputation strategies under the scenario that all informative people have dense genotypes for family-based imputation and all individuals have sparse genotype as the backbone for population-based imputation, using genotypes simulated from haplotype pool 2 (all UK10K sample). The x-axis in the figures is on a logarithmic scale.

**Figure 4:**
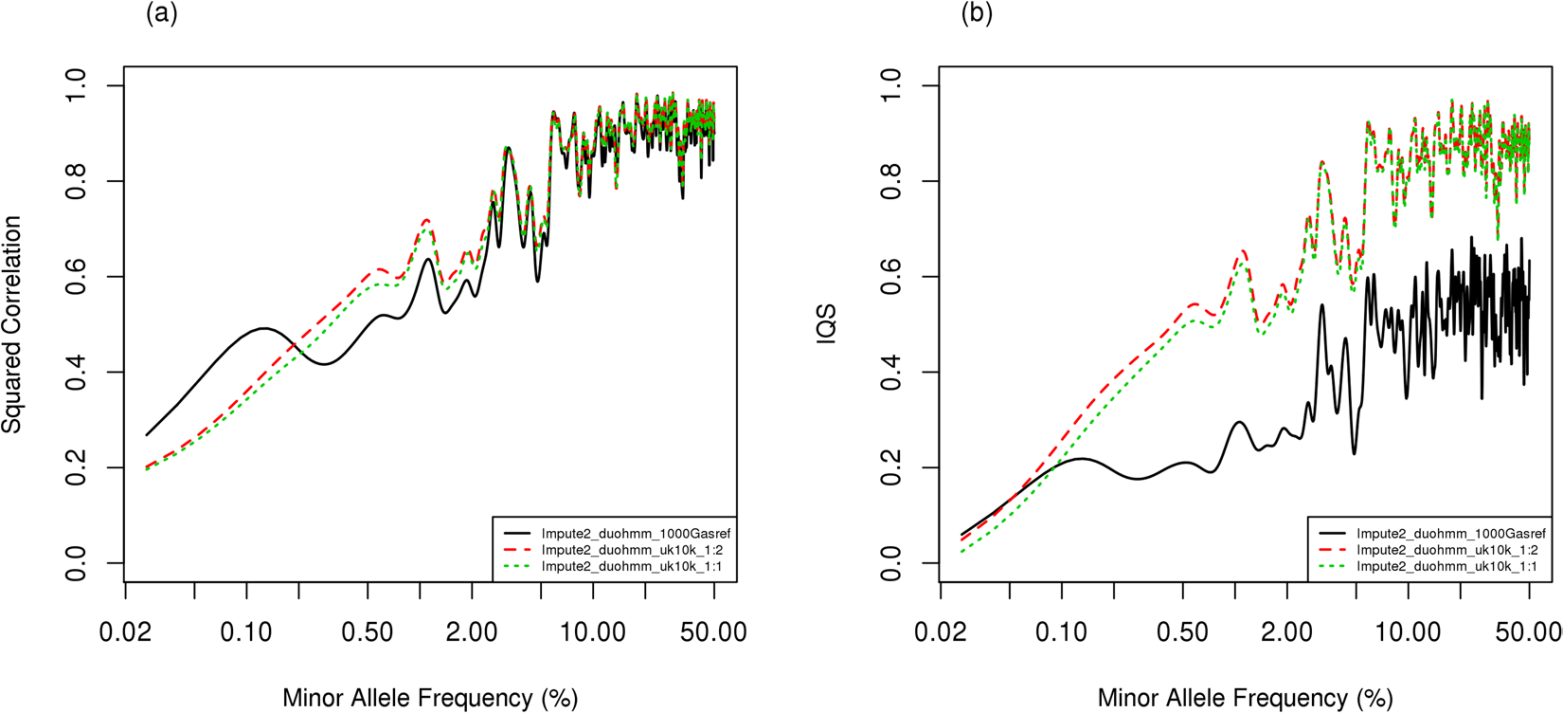
Comparison of imputation accuracy with squared correlation, (**a**) and imputation quality score, (**b**) among various population-based imputation strategies under the scenario that all individuals have sparse genotypes as the backbone for population-based imputation, using genotypes simulated from haplotype pool 3 (1/3 of UK10K sample). The x-axis in the figures is on a logarithmic scale.

As described, genotypic data were generated from three sources (H1: UK10K + 1KG, H2: UK10K only; H3: 1/3 of UK10K); imputation was performed using five imputation methods (I1: Impute2_unrelated, I2: Impute2_duohmm, I3: GIGI, I4: MERLIN and I5: PedBLIMP) along with four reference panels (R1: UK10K + 1KG, R2: 1KG, R3: 1/3 of UK10K, R4: 2/3 of UK10K) for imputation (Fig. 1). The squared correlation and IQS measured the agreement with true simulated genotypes. The results are displayed in Figs 2, 3 and 4.

#### Ideal scenario for population-based imputation

Here the haplotype information in the reference panel for population-based imputation came from the same source of the haplotype information for genotypic data generation, i.e. H1 (Fig. 1a). An unlikely situation, it showed how good PBI can be using an extremely good reference panel. As expected, Impute2_duoHMM performed excellently with both squared correlation (*r*^2^) and IQS close to 1 (Fig. 2). In the scenario with family size 12 (Fig. 2a,b), both MERLIN and GIGI showed comparable imputation quality to Impute2_duoHMM for variants with MAF <5%. However, for variants with MAF >5%, the imputation quality for both MERLIN and GIGI was lower although their performance remained high with both *r*^2^ and IQS greater than 0.994. The imputation approach without utilizing pedigree structure information (Impute2_unrel) performed worst even with the ideal reference panel except for variants with MAF greater than 20% where it slightly outperformed FBI. The variability of the estimated accuracy was larger in Impute2_unrel than other approaches. However, variabilities were consistently low, especially for MERLIN and GIGI. Hence, variability information is not provided hereafter. We also evaluated a larger pedigree of size 40, selected from the Framingham Heart Study, where MERLIN requires splitting the whole pedigree into sub-pedigrees. We evaluated two options for maxbit: 18 and 20. MERLIN with maxbit 18 retains imputation quality as good as GIGI while there is a clear drop in imputation quality for MERLIN with maxbit 20 (Fig. 2c,d). This comparison indicates that different pedigree splits can affect imputation performance dramatically.

#### Comparison among imputation approaches: family-based, population-based and hybrid imputation

Next, we evaluated imputation performance with family size 12 by considering different sources of haplotype information between genotypic data generation (UK10K) and the reference panel (1000 G) (Fig. 1b). In addition to comparing traditional PBI and FBI, we include a newly proposed hybrid approach, PedBLIMP. For both the squared correlation and IQS, FBI (MERLIN and GIGI) performs best, followed by PedBLIMP, and PBI (Impute2_duoHMM) shows the worst performance. Interestingly, the performance of PedBLIMP drops but that of Impute2_duoHMM increases when the MAF of imputed variants >5% for IQS (Fig. 3b).

#### Effect of different sources and sizes of reference panel for population-based imputation

We further evaluated the imputation accuracy using different reference panels (Fig. 1c). The haplotype information from UK10K was randomly divided into three groups with each having 1260 subjects. From them, one group was randomly selected to serve as the haplotype pool (H3) for data generation. The reference panel then used a randomly selected one of the two remaining thirds (R3) or all remaining haplotype data (R4). In addition, we evaluated 1000 G (R2) as the reference panel. For both imputation accuracy measures, using 1/3 of UK10K (R3) and 2/3 of UK10K (R4) performed similarly, although the larger reference panel yields slightly better performance for rare or less frequent variants (Fig. 4). For IQS, 1/3 of UK10K (R3) and 2/3 of UK10K (R4) performed better than 1000 G (R2), indicating better imputation accuracy with a closely related population as a reference panel (Fig. 4b).

#### Family-based Imputation of first generation individuals who do not have genetic information

It is not uncommon that DNA samples are unavailable for an earlier generation while other clinical variables are available for analysis. Including these individuals’ imputed genotypes will increase the sample size and power for downstream analysis. We evaluated the imputation results for the first generation individuals using the second and third generations’ genotype information (Supplemental Fig. 2) with MERLIN and GIGI, as PBI was not applicable. In general, GIGI outperformed MERLIN for both the squared correlation and IQS accuracy measures. The only exception was that higher IQS values were observed for MERLIN for variants with minor allele frequency less than 1% (bottom panel of Supplemental Fig. 2).

## Discussion

We conducted intensive simulation to evaluate the performance of several strategies to impute missing genotypes. Our data demonstrated that FBI, specifically MERLIN and GIGI, provides better imputation quality in general when there are informative family members with available dense sequence data, while PBI, specifically Impute2_duoHMM, offers reasonable imputation quality for common variants especially when a closely related reference panel is used. In addition, MERLIN and GIGI produced imputation quality comparable to each other in small pedigrees; however, as a cautious point, MERLIN can only handle pedigrees of small or moderate size and requires splitting of large pedigrees, which has an observable effect on imputation accuracy. A separate effort to comprehensively describe the properties of MERLIN, especially the strategy on the splitting of large pedigrees, is needed.

PBI has facilitated identification of common variants associated with complex diseases; however much genetic variation remains to be explained and less frequent variants may contribute. Various imputation approaches have been proposed to enhance imputation accuracy of rare variants. The growing size of available reference panels will provide better coverage of longer stretches of haplotypes^27,28^. However, we still observed an apparent gap in the imputation accuracy compared to family-based imputation. Other studies suggest using a local reference or study-specific samples^29,30^, consistent with our observation in the ideal scenario for population-based imputation, an unusual case, where the haplotype sources are the same for target study samples and reference panel. The advantage for this type of approach comes from the potential population-specific haplotypes introduced by low frequency variants and a local reference panel can provide more precise haplotypes than publicly available reference panels of similar size. The information used for FBI shares similar merit as a local reference panel. However, the advantage of FBI is less likely to diminish even when publicly available reference panels grow with dense sequencing of more individuals as such an ideal scenario for population-based imputation may not be realistic. Additionally, imputation to family members, such as older generations, without genotype information is only applicable in a family-based approach.

Leveraging the information from LD and inheritance patterns may improve genotype imputation. There may not always be enough family members with dense sequence data to obtain accurate inheritance vectors, although the family-based imputation clearly dominates the imputation accuracy. LD information from large publicly available genotype resources can provide imputation for common variants with a well-selected reference panel without requiring densely sequenced data in family members. Therefore, methods to use the strengths from both PBI and FBI may provide alternative strategies. Indeed, Saad and Wijsman^31,32^ showed that combining PBI and FBI of sequence data improves statistical power to detect rare variant associations. Lent *et al*.^33^ demonstrated that a sequential approach that uses PBI to increase the density of a sequenced reference panel before performing FBI further increases imputation accuracy. Results that are more powerful were observed through such approaches, but these only used information from LD and inheritance patterns indirectly. Recently, PedBLIMP^14^ uses both pedigree structure and LD information to define the genotype covariance matrix used in a linear prediction model. As observed in our simulation, the improvement of the imputation accuracy with this approach compared to the population-based imputation is obvious although there remains room for improvement compared to the family-based imputation.

Our study comes with several strengths and limitations. Among the strengths, first, we used UK10K plus 1000 G, the largest publicly available haplotype reference panel, at the time we started this project. The release of more dense reference panels will continue to improve PBI. Second, we conducted extensive simulations to systematically evaluate the accuracy of genotype imputation for FBI, PBI and hybrid strategies. Third, imputation to the earlier generation of individuals based on the available data in later generations was also evaluated. This evaluation provided useful information as it is relatively common to lack DNA data in earlier generations due to death or loss to follow up.

Some limitations of our study include the fact that we primarily simulated a specific pedigree structure with three generations. This structure may provide more information in imputation than a trios study design but is less informative than more extended family pedigrees, such as family structures collected in the Framingham Heart Study. Larger pedigrees may affect the imputation accuracy for family-based imputation. However, MERLIN cannot be used in large pedigrees without splitting. We demonstrated this approach in one of our simulation settings. On the other hand, we assumed an ideal scenario that all informative individuals within the pedigree have dense genotypes, a circumstance that may not always be practical for various reasons such as cost or lack of DNA samples for genotyping. Considering information from related individuals, the imputation results and their downstream association analysis will depend on the selection of subjects to densely genotype. Third, to better evaluate imputation performance for rare variants, we conducted extensive simulations in a specific genomic region rather than simulating whole genome data, due to the computational constraints. However, the selected region has a wide range of allele frequencies and various LD structures; so we do not anticipate substantial changes in our findings should the simulation be done in the whole genome.

Imputation methods provide information on the distribution of possible genotypes at untyped variants for very little cost compared to direct genotyping or sequencing. As observed in our study, family-based imputation when informative individuals within a pedigree having dense genotypes outperformed population-based imputation even with haplotype phasing estimated using family relationships. This advantage was found in both common and LF variants while population-based imputation may provide relatively comparable results for common variants if closely ancestry-matched samples are available for the reference. Combining family- and population-based imputation data may provide an alternative option to improve imputation quality as suggested by Saad and Wijsman^32^ where better imputed genotypes for each variant from either family-based or population-based imputation are used. Hence, an approach that can simultaneously use LD information from a large haplotype reference and extra information from related subjects may provide fruitful results in genotype imputation and identification of risk variants underlying complex disease.

## Supplementary information

Supplemental Figures 1 and 2
