# Supplementary material for: Revisit Population-based and Family-based Genotype Imputation
Source: Sci Rep. 2019 Feb 12;9:1800. doi: 10.1038/s41598-018-38469-4 (PMC6372660; doi:10.1038/s41598-018-38469-4)

Revisit Population-based and Family-based Genotype Imputation

Ching-Ti Liu[1], Xuan Deng[1], Virginia Fisher[1], Nancy Heard-Costa[2], Hanfei Xu[1], Yanhua Zhou[1], Ramachandran S. Vasan[2,3], L Adrienne Cupples[1,3]

[1] Department of Biostatistics, Boston University School of Public Health, Boston MA 02118

[2] Department of Medicine, Boston University, Boston MA 02118

[3] Framingham Heart Study, Framingham MA 01702

Corresponding authors:

Ching-Ti Liu, Ph.D.

Associate Professor, Department of Biostatistics, School of Public Health, Boston University,

801 Massachusetts Ave CT3, Boston, MA 02118, USA

Tel: +1 6176387752

Fax: +1 6176386484

Email: ctliu@bu.edu

L Adrienne Cupples, Ph.D.

Professor, Department of Biostatistics, School of Public Health, Boston University,

801 Massachusetts Ave CT3, Boston, MA 02118, USA

Tel: +1 6176385176

Fax: +1 6176386484

Email: adrienne@bu.edu

Supplemental Figure 1. Pedigree structure and informative individuals for pedigree of size 12.

Supplemental Figure 2. Comparison of imputation accuracy with squared correlation, **Supplemental Figure 2a**, and imputation quality score, **Supplemental Figure 2b**, among various imputation strategies under the scenario that genotype information for the first generation was completely imputed based on the second and third generations' genotype information for 1000 families of size 12. The x-axis in the figure is on a logarithmic scale.

Supplemental Figure 1

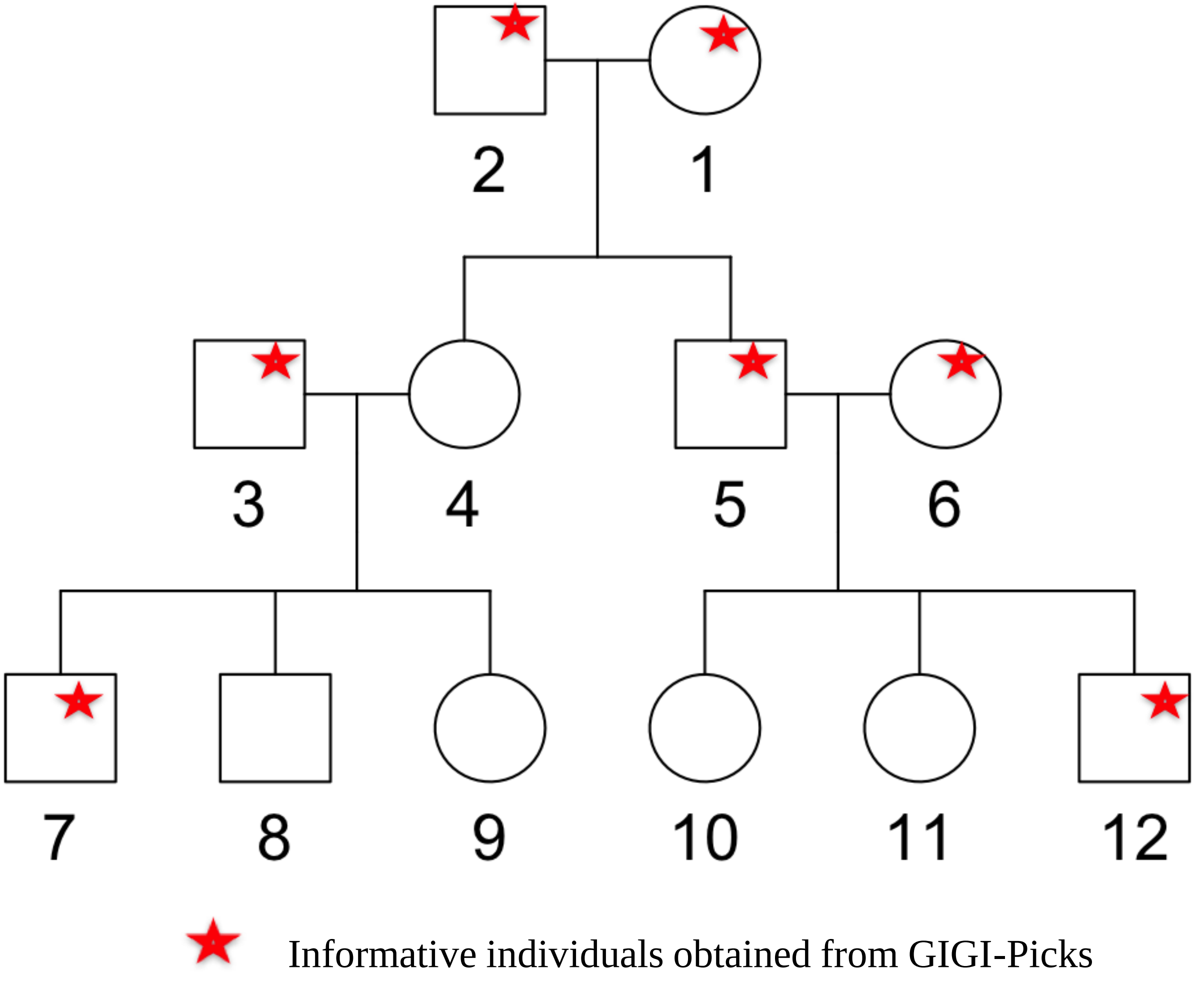

Supplemental Figure 2

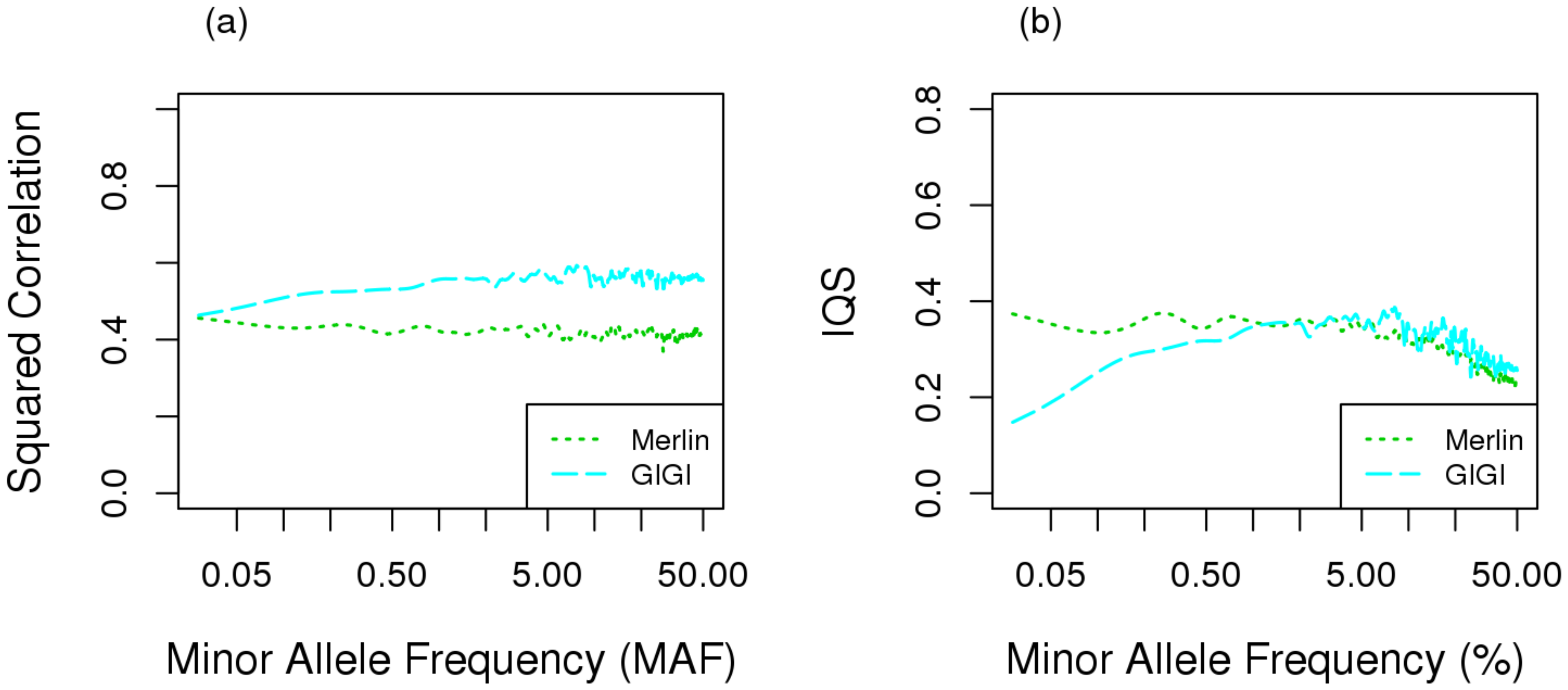

Supplement: Supplementary file 1 — Supplemental Figures 1 and 2 [file 41598_2018_38469_MOESM1_ESM.pdf]
